# Predicting type 2 diabetes mellitus: a comparison between the FINDRISC score and the metabolic syndrome

**DOI:** 10.1186/s13098-018-0310-0

**Published:** 2018-03-01

**Authors:** Abraham S. Meijnikman, Christophe E. M. De Block, An Verrijken, Ilse Mertens, Luc F. Van Gaal

**Affiliations:** Department of Endocrinology, Diabetology & Metabolism, Faculty of Medicine, Antwerp University Hospital and University of Antwerp, Wilrijkstraat 10, 2650 Edegem, Belgium

**Keywords:** Type 2 diabetes mellitus, Prediabetes, Screening, Metabolic syndrome, FINDRISC, Obesity

## Abstract

**Background:**

The aim of this study to compare the diagnostic accuracy of the metabolic syndrome (MetS) with the FINDRISC score to screen for type 2 diabetes mellitus T2DM in an overweight/obese population.

**Methods:**

Subjects 18 years or older visiting the obesity clinic of the Antwerp University Hospital were consecutively recruited between 2012 and 2014. Every patient underwent a standard metabolic work-up including a clinical examination with anthropometry. Glucose status was tested using OGTT and Hba1c. FINDRISC questionnaire and MetS were examined.

**Results:**

Of 651 subjects, 50.4% were diagnosed with prediabetes, whereas 11.1% was diagnosed with T2DM. FINDRISC score increased with worsening of glucose status 11 ± 3, 13 ± 4 and 15 ± 5 in respectively, subjects without T2DM, prediabetes and T2DM. 312 subjects had the MetS. The aROC of the FINDRISC to identify subjects with T2DM was 0.76 (95% CI 0.72–0.82), sensitivity was 64% and specificity was 63% with 13 as cutoff point. Adding FPG or HbA1c to FINDRISC, the aROC increased significantly to 0.91(95% CI 0.88–0.95) and 0.93(95% CI 0.90–0.97), respectively (p < 0.001). The aROC of the MetS to identify subjects with diabetes was 0.72 (95% CI 0.65–0.78), sensitivity was 75% and specificity was 55%. The aROC of the FINDRISC + HbA1c was significantly higher than the MetS for predicting T2DM (p < 0.001).

**Conclusion:**

Prediction of type 2 diabetes is important for timely intervention and to avoid chronic complications associated with the disease. Our findings suggest, that it may be of good clinical practice to use the FINDRISC score + HbA1c in a two-step screening model for diabetes rather than using the metabolic syndrome.

## Background

According to the International Diabetes Federation, it is estimated that every 6 s one person dies from diabetes and more than 600 billion USD is spent on diabetes related healthcare [1]. Despite several prevention programs and attempts to create global awareness of the risks of type 2 diabetes, the prevalence is expected to increase to 642 million in 2040 [1]. Due to the human and economical burden of the disease and its complications, there is a tremendous need for a cost efficient, accurate and feasible screening tool for type 2 diabetes. In the majority of the subjects with prediabetes or type 2 diabetes, a set of risk factors commonly appears together, which is now known as the metabolic syndrome. The metabolic syndrome, which is a cluster of insulin resistance and disturbed glucose metabolism, overweight and abdominal fat distribution, dyslipidemia and high blood pressure, is an important determinant of cardiovascular risk [2]. It is estimated that about 25% of the world adult population has the metabolic syndrome [3] In addition, subjects with the metabolic syndrome have a 5 times higher risk of developing type 2 diabetes [4–7]. However, before the diagnosis of the metabolic syndrome can be established, five clinical and biochemical parameters need to be assessed, making it one of the most labor intensive and cost expensive prediction tools for type 2 diabetes mellitus [8].

The Finnish Diabetes Risk Score (FINDRISC) is another commonly used and validated non-invasive diabetes risk score worldwide and has proven its value in the national type 2 diabetes prevention program in Finland [9]. The risk score consists of eight non-invasive clinical characteristics such as age, body mass index (BMI), waist circumference, daily physical activity, eating habits, blood pressure or antihypertensive medication, a history of high blood glucose and family history of diabetes.

However, to the best of our knowledge, the discriminatory accuracy of the metabolic syndrome versus the FINDRISC score has never been tested before. Therefore, we compared the metabolic syndrome to the FINDRISC score as a screening tool for type 2 diabetes mellitus in a population of apparently healthy overweight and obese subjects without a prior diagnosis of diabetes, considered as an ‘at risk’ population.

## Methods

Subjects 18 years or older visiting the obesity clinic of the Antwerp University Hospital (a tertiary referral facility) for a problem of overweight (BMI ≥ 25–29.9 kg/m^2^) or obesity (BMI ≥ 30 kg/m^2^) were consecutively recruited between 2012 and 2014, after written informed consent. Subjects with a history of diabetes or those already taking anti-diabetic medication and non-Caucasian subjects were excluded.

Every patient underwent a standard metabolic work-up including a clinical examination with anthropometry. All measurements were performed in the morning, with patients in fasting conditions and undressed. Height was measured to the nearest 0.5 cm using a wall-mounted stadiometer, and body weight was measured using a digital scale to the nearest 0.2 kg with subjects in their underwear and without shoes. Body mass index (BMI) was calculated as weight in kilograms over height in square meters. Waist circumference was measured at the mid-level between the lower rib margin and the iliac crest. Visceral abdominal adipose tissue (VAT) and subcutaneous abdominal adipose tissue (SAT) were determined by a 64-slice computerized tomography (CT) scan at the L4–L5 level (slice thickness 0.6 mm), according to the technique described by Van der Kooy and Seidell [10] and Kvist [11]. First, the total area of abdominal tissue was measured at −190 to 330 HU. Subsequently, the surface area of different fat compartments was assessed using a dedicated software (AW VolumeShare 2). The total adipose tissue area was calculated in the area limited by the skin including the epidermis. Visceral adipose tissue was calculated in the area within the fascia transversalis. The difference between the total fat tissue and the visceral fat was equivalent to the subcutaneous fat tissue.

Fasting and a 2-h oral glucose tolerance test, using 75-g glucose, was performed, and insulin and c-peptide levels were analyzed. Insulin resistance and beta cell function was estimated using homeostasis model assessment (HOMA) as described by Matthews et al. [12]. Insulin resistance (HOMA-IR) was calculated as [insulin (mU/l) × glucose (mmol/l)]/22.5, with one as reference value for normal insulin sensitivity. Beta cell function (HOMA-B) was calculated as 20 × (insulin (mU/l)/(glucose (mmol/l)—3.5).

### Diabetes and pre-diabetes definitions

The American Diabetes Association classification for the diagnosis of prediabetes and diabetes was used [13]. Subjects who had a fasting glucose (FPG) ≥ 126 mg/dl and/or a HbA1c ≥ 6.5% and/or a 2-h plasma glucose ≥ 200 mg/dl were classified as de novo diabetes. Subjects with an impaired fasting glucose (IFG) (100–125 mg/dl) or/and an impaired glucose tolerance (IGT) (140–199 mg/dl) or/and a HbA1c between 5.7 and 6.4% were classified as being pre-diabetic.

### Metabolic syndrome criteria

The metabolic syndrome was evaluated according to the National Cholesterol Education Program Adult Treatment Panel III (NCEP-ATP III) [8], subjects were classified as having the metabolic syndrome if ≥ three of the following criteria were met: (1) abdominal obesity (waist circumference ≥ 102 cm in men and ≥ 88 cm in women), (2) hypertriglyceridemia (≥ 150 mg/dl), (3) low HDL cholesterol (< 40 mg/dl in men and < 50 mg/dl in women), (4) high blood pressure (≥ 130/85 mmHg) or on anti-hypertensive medication, (5) high fasting plasma glucose (≥ 100 mg/dl).

### Laboratory measurements

HbA1C was determined by high performance liquid chromatography (Adams™ A1c HA-8160, Arkray–Menarini instrument, Zaventem, Belgium; reference range: 4.8–6.0%). Plasma levels of glucose, creatinin, total cholesterol and triglycerides were measured on Vitros 750 XRC (Ortho Clinical Diagnostics, Johnson & Johnson, Buckinghamshire, UK). HDL-C was measured on Hitachi 912 (Roche Diagnostics, Mannheim, Germany). Insulin levels were measured with the Medgenic two-site IRMA assay (BioSource, Belgium). Microalbuminuria was measured with nephelometry using a Behring Nephelometer II (Siemens Healthcare Diagnostics Products, Marburg, Germany).

### Statistical methods

SPSS version 23 was used for statistical analyses. Data are expressed as mean ± SD for normally distributed variables or as median (interquartile range) when distribution is skewed. The normal distribution of continuous variables was assessed with the Kolmogorov–Smirnov method. Comparison of population characteristics was performed with an independent samples t test or a Mann–Whitney U test, depending on normality. Variables, which were not normally distributed, were log transformed. The receiver-operating characteristic (ROC) curves were constructed to show the relationship between the sensitivity and specificity of the FINDRISC score for identifying subjects with type 2 diabetes. The area under the receiver-operating curve (aROC) was used to evaluate the discriminatory accuracy of the FINDRISC to identify prediabetes and diabetes subjects. An aROC of 1.0 indicates a perfect test with no false positive rate and no false negative rate, an aROC of 0.5 indicates that the test performed not better than chance. The cutoff points to identify prediabetes and diabetes were determined by the point with the shortest distance to the upper left corner in the ROC curve, which was calculated as the square root of [(1-sensitivity) [2] + (1-specificity) [2] ]. The same statistical analysis was performed to evaluate discriminatory accuracy of the metabolic syndrome to identify subjects with type 2 diabetes mellitus.

## Results

In total, 651 (Male/female: 193/458) overweight and obese subjects, with an average age of 43 ± 13 year were examined. Baseline characteristics are presented in Table 1. Exactly 187 subjects were already on anti-hypertensive medication and 76 were on lipid lowering medication (either statin or fibrate).

**Table 1:** Baseline characteristics of 651 overweight or obese subjects without a history of diabetes

	All	Men	Women	P
Number of cases	651	193	458	
Age (years)	43 ± 13	46 ± 12	42 ± 13	0.03
Weight (kg)	109.3 ± 21.2	124.5 ± 22.1	102.4 ± 17.2	< 0.01
BMI (kg/m^2^)	38.2 ± 6.1	39.0 ± 6.4	37.9 ± 5.9	0.02
Waist circumference (cm)	116.2 ± 14.8	126.3 ± 12.6	112 ± 13	< 0.01
Systolic BP (mmHg)	129 ± 15	133 ± 14	127 ± 15	0.02
Diastolic BP (mmHg)	76 ± 11	80 ± 11	75 ± 10	0.03
Triglycerides (mg/dl)	132 (97–185)	163 (114–220)	125 (91–170)	< 0.01
Total cholesterol (mg/dl)	198 (175–227)	189 (167–219)	203 (177–230)	< 0.01
HDL cholesterol (mg/dl)	49 (40–60)	42 (35–50)	52 (44–63)	< 0.01
CT visceral fat (cm^2^)	183 (131–249)	249 (194–315)	157 (113–209)	< 0.01
CT subcutaneous fat (cm^2^)	593 ± 145	562 ± 167	605 ± 132	< 0.01
CT total fat (cm^2^)	789 ± 173	820 ± 189	775 ± 164	< 0.01
Fasting glucose (mg/dl)	86 (80–95)	92 (84–101)	84 (79–92)	0.01
2-h OGTT glucose (mg/dl)	140 (118–167)	147 (125–185)	138 (117–161)	< 0.01
HbA1c (%)	5.5 (5.3–5.8)	5.6 (5.4–6.0)	5.4 (5.2–5.7)	0.03
HbA1c (mmol/mol)	37 (34–40)	38 (36–41)	36 (33–37)	0.03
FINDRISC score	12 ± 4	13 ± 4	12 ± 4	0.54
HOMA-S	3.3 (2.1–5.4)	4.5 (2.9–7.0)	3.0 (1.8–4.6)	< 0.01
HOMA-B (%)	243 (153–375)	241 (153–361)	247 (157–380)	0.65
Microalbuminuria (μg/min)	8.0 (5.9–13.0)	9.0 (7.0–16.0)	8.0 (5.0–12.0)	0.01

Data are expressed as mean ± standard deviation or median (range)

*BMI* body mass index, *BP* blood pressure, *HDL* high density lipoprotein, *OGTT* oral glucose tolerance test, *HOMA-S* homeostasis model assessment of insulin resistance, *HOMA-B* homeostasis model assessment of beta-cell function

According to the American Diabetes Association diagnostic criteria, 72 patients were newly diagnosed with type 2 diabetes mellitus, 328 were classified with prediabetes and 251 were considered non-diabetic. In total, 312 subjects met the diagnostic criteria for metabolic syndrome using the NCEP-ATP III criteria. The FINDRISC score increased with worsening of glucose tolerance. In subjects without type 2 diabetes, the FINDRISC score was 11 ± 3, in subjects with prediabetes 13 ± 4 and in subjects with type 2 diabetes mellitus 15 ± 5. The proportion of subjects with the metabolic syndrome also increased with worsening of the glucose tolerance, 32% in subjects without diabetes, 54% in subjects with prediabetes and 75% in subjects with de novo diagnosed diabetes according to the NCEP-ATP III criteria. Similar results were observed when using other criteria for the metabolic syndrome, such as the World Health Organisation criteria and the Harmonized criteria.

The discriminatory accuracy of the FINDRISC for identifying subjects with diabetes, assessed by the aROC was 0.76 (95% CI 0.72–0.82). The cutoff point of the FINDRISC score for detecting diabetes with the shortest distance to the upper left corner of the ROC plot proved to be a FINDRISC score of 13 with a sensitivity of 64% and specificity of 63% as previously reported by our group [8]. The positive predictive value was 18% and the negative predictive value was 93%. When adding FPG or HbA1c to the original FINDRISC score to identify diabetes, the aROC significantly increased to 0.91(95% CI 0.88–0.95) and 0.93(95% CI 0.90–0.97), respectively (p < 0.001).

Of the 312 subjects who were diagnosed with metabolic syndrome, 151 had a FINDRISC score > 13 when using the NCEP-ATP III criteria.

The aROC of the FINDRISC to identify subjects with the metabolic syndrome according to the NCEP-ATP III criteria was 0.69 (95% CI 0.64–0.75). The discriminatory accuracy of the metabolic syndrome assessed by the aROC for identifying subjects with diabetes was 0.72 (95% CI 0.65–0.78) with a sensitivity of 75% and specificity of 55%. The positive predictive value was 17% and the negative predictive value was 95%.

The aROC of the original FINDRISC score did not differ significantly to the aROC of the metabolic syndrome to identify subjects with diabetes. However, by adding FPG or HbA1c, the discriminatory accuracy of the model did differ significantly compared to the metabolic syndrome (p < 0.001).

Characteristics of subjects with a FINDRISC score < 13 corresponds with the characteristics of the subjects without the metabolic syndrome and the same applies for subjects with a FINDRISC score ≥ 13 and subjects with the metabolic syndrome (see Table 2).

**Table 2:** Comparison between subjects with a FINDRISC score < 13 vs subjects without the metabolic syndrome and subjects with a FINDRISC ≥ 13 vs subjects with the metabolic syndrome

	FINDRISC < 13	Metabolic healthy	P1	FINDRISC ≥ 13	Metabolic syndrome	P2
Number of cases	389	339		262	312	
Age (years)	40 ± 12	41 ± 13	0.89	47 ± 12	45 ± 12	0.83
Weight (kg)	108.3 ± 22.0	104.7 ± 20.3	0.32	110.9 ± 19.9	114 ± 20.9	0.02
BMI (kg/m^2^)	37.8 ± 6.2	37.5 ± 6.2	0.91	38.9 ± 5.8	39.0 ± 5.8	0.85
Waist (cm)	113.9 ± 14.9	113.8 ± 15.6	0.98	119.4 ± 13.3	120.8 ± 13.2	0.88
Systolic BP (mmHg)	126 ± 15	123 ± 13	0.76	132 ± 14	134 ± 14	0.82
Diastolic BP (mmHg)	75 ± 11	73 ± 10	0.89	78 ± 11	80 ± 11	0.79
Triglycerides (mg/dl)	125 (90–177)	106 (80–130)	< 0.001	150 (108–198)	179 (150–1229)	0.01
Cholesterol (mg/dl)	196 (175–226)	197 (172–219)	0.86	202 (173–229)	201 (175–235)	0.87
HDL cholesterol (mg/dl)	48 (40–60)	56 (48–66)	< 0.001	49 (40–60)	42 (40–60)	0.05
CT visceral fat (cm^2^)	169 (115–225)	155 (102–212)	< 0.001	206 (148–277)	210 (158–267)	0.20
CT subcutaneous fat (cm^2^)	592 (592)	601 (149)	0.10	593 (138)	584 (137)	0.06
Total fat (cm^2^)	773 ± 175	771 ± 183	0.68	812 ± 166	806 ± 159	0.52
Fasting glucose (mg/dl)	84 (79–91)	83 (78–91)	0.84	90 (83–99)	92 (82–101)	0.83
2-h OGTT glucose (mg/dl)	133 (114–154)	131 (113–155)	0.79	155 (129–186)	152 (150–184)	0.68
Hba1c (%)	5.4 (5.2–5.6)	5.5 (5.2–5.6)	0.56	5.6 (5.4–5.9)	5.6 (5.4–5.9)	0.91
HbA1c (mmol/mol)	36 (33–38)	36 (33–38)	0.97	38 (36–41)	38 (36–40)	0.98
Microalbuminuria (μg/min)	8.0 (5–11)	11 (5–12)	0.43	10.0 (6–15)	9.0 (6–15)	0.66
FINDRISC score	10 ± 2	11 ± 4	0.74	16 ± 3	13 ± 4	0.03
HOMA-S	2.9 (1.9–4.8)	2.7 (1.8–4.2)	0.63	3.9 (2.5–6.0)	4.2 (2.7–6.0)	0.04
HOMA-B (%)	252 (164–392)	233 (152–369)	< 0.03	236 (147–337)	261 (165–385)	< 0.01
Metbalic syndrome	228/161			111/151		

Data are expressed as mean ± standard deviation or median (range)

*BMI* body mass index, *BP* blood pressure, *HDL* high density lipoprotein, *OGTT* oral glucose tolerance test, *HOMA-S* homeostasis model assessment of insulin resistance, *HOMA-B* homeostasis model assessment of beta-cell function, *P1* P value for the comparison between FINDRISC < 13 and Metabolic Healthy, *P2* P value for the comparison between FINDRISC ≥ 13 and metabolic syndrome

### Correlation analysis

Univariate analyses showed significant correlations between the FINDRISC score and HOMA-IR (r = 0.24, p < 0.001), HOMA-B (r = − 0.09, p = 0.021), HbA1c (r = 0.35, p < 0.001), FPG (r = 0.34 p < 0.001), 2 h OGTT glucose (r = 0.34, p < 0.001), VAT (r = 0.34, p < 0.001), VAT/SAT ratio (r = 0.39, p < 0.001) and microalbuminuria (r = 0.12, p = 0.002).

The metabolic syndrome was significantly correlated with the FINDRISC score (r = 0.24, p < 0.001), HOMA-IR (r = 0.31, p < 0.001), VAT (r = 0.33, p < 0.001), VAT/SAT ratio (r = 0.31, p < 0.001), HbA1c (r = 0.22, p < 0.001), FPG (r = 0.27, p < 0.001), 2-h OGTT glucose (r = 0.28, p < 0.001) and microalbuminuria (r = 0.18, p < 0.001).

## Discussion

Prediction of type 2 diabetes is important for timely intervention and to avoid chronic complications associated with the disease. To the best of our knowledge, this is the first study comparing the discriminatory accuracy of the metabolic syndrome versus the FINDRISC score to predict type 2 diabetes. The ability of the FINDRISC to identify subjects with type 2 diabetes mellitus in an overweight and obese population has been shown before [14]. The metabolic syndrome showed a relative good ability to identify subjects with type 2 diabetes according to an aROC of 0.72 with a sensitivity of 75% and specificity of 55%, the positive predictive value was 17% and the negative predictive value 95%. However, the aROC of the FINDRISC did not differ significantly compared to the aROC of the metabolic syndrome. Since FPG is one of the components that need to be tested for the metabolic syndrome, we investigated whether adding FPG to the FINDRISC questionnaire would improve its discriminatory accuracy. We observed a significant increase in discriminatory accuracy to 0.91 when FPG was added to the original FINDRISC score. When HbA1c was added instead of FPG, it increased to 0.93. Others previously reported that the performance of a diabetes risk score could be improved by adding biochemical markers. Adding lipids and FPG to the Atherosclerosis Risk in Communities (ARIC) study, the aROC increased from 0.71 to 0.80 (p < 0.001) [15]. In the Framingham Offspring Study, the aROC increased from 0.72 to 0.85 when FPG, HDL-cholesterol and triglycerides were added to the original risk score [16]. When FPG, triglycerides, HbA1c, HDL-cholesterol and liver enzymes were added to the German Diabetes RISK score, the aROC increased from 0.85 to 0.90 (ref: [17]). More sophisticated tests, such as the estimated insulin resistance (HOMA-IR) or estimated insulin secretion (HOMA-B) did not significantly improve the discriminatory accuracy of the Framingham Offspring Study [16]. In contrast, the San Antonio Heart Study model did improve significantly when adding an OGTT, 1-h plasma glucose or HOMA-IR or HOMA-B [18, 19].

However, including FPG or HbA1c concentrations into the model introduces a bias, since you introduce parameters, which are used for the diagnosis of diabetes itself, thereby falsely increasing the discriminative power of such a risk score. Moreover, the introduction of parameters, which are used for the diagnosis of diabetes itself, defeats the whole point of why clinicians should use diabetes risk scores. However, the same is true for the metabolic syndrome. Nevertheless, it is worth mentioning that a non-invasive screening tool combined with a relative inexpensive and feasible biochemical maker is a better tool to identify subjects with diabetes than a cluster of five clinical and biochemical makers, which are needed to diagnose the metabolic syndrome.

The prevalence of the metabolic syndrome increased with increasing FINDRISC score. Several cross-sectional studies have assessed the FINDRISC score as a screening tool for the metabolic syndrome [20, 21]. A Greek study evaluated a simplified FINDRISC score to identify subjects with the metabolic syndrome and found an aROC of 0.71 and 0.76 in men and women, respectively [20] Saaristo et al. [21] examined the prevalence of metabolic syndrome in a Finnish population and found an aROC of 0.73 in men and 0.75 in women. The aROC of the FINDRISC score to identify subjects with the metabolic syndrome in this study was lower than those in other cross-sectional studies. In a prospective study, the FINDRISC showed a reasonable ability to rightfully predict if a subject develops the metabolic syndrome, with an aROC of 0.65 ref [22].

This is the first study where the FINDRISC score and the metabolic syndrome are compared to identify subjects with type 2 diabetes mellitus. A major strength of this study is that it consists of 651 well-characterized subjects, and this can be considered as a large cohort. Another strength is that the FINDRISC questionnaire was taken by a health care professional, thereby limiting inaccuracy and increasing reliability.

A relative limitation is the small number of men analyzed in this study. The subjects included in this study were referred by their general practitioner or came at their own initiative and it is well known that women tend to seek help for medical problems more often and earlier than men. Nevertheless, the number of men are in our opinion sufficient enough to generalize these study results. However, validation of these results in other (i.e. larger) cohorts is desirable. Another limitation is the cross-sectional study nature of this study, precluding causality. It would be interesting to organize follow-up to improve the prediction model, not only for diabetes status but also cardiovascular outcome. Data collection continues as we report.

## Conclusion

In a population consisting of only subjects with overweight or obesity, 72 subjects were newly diagnosed with type 2 diabetes mellitus and 328 were diagnosed with prediabetes. In total, 312 subjects met the diagnostic criteria for the metabolic syndrome. Of these 312 subjects, 151 had a FINDRISC score > 13 using the NCEP-ATP III criteria.

The discriminatory accuracy to identify subjects with diabetes of the original FINDRISC questionnaire was similar to that of the metabolic syndrome, but much easier to perform since it does not require invasive testing. However, when adding Hba1c or FPG the FINDRISC score outperformed the metabolic syndrome. Therefore, our findings suggest, that it may be of good clinical practice to use the FINDRISC score + HbA1c (with the official cutoff point for diabetes) in a two-step screening model for diabetes rather than using the metabolic syndrome.

## Abbreviations

MetS: metabolic syndrome
T2DM: type 2 diabetes mellitus
OGTT: oral glucose tolerance test
BMI: body mass index
HOMA-IR: homeostasis model assessment for insulin resistance
HOMA-B: homeostasis model assessment for beta cell function
NCEP-ATP III: National Cholesterol Education Program Adult Treatment Panel III
FPG: fasting plasma glucose
IFG: impaired fasting glucose
IGT: impaired glucose tolerance
HDL: high-density lipoprotein
aROC: the area under the receiver-operating curve
